# Incidence, prevalence, mortality, and causes of death in Waldenström macroglobulinemia: a nationwide, population-based cohort study

**DOI:** 10.1186/s12885-020-07120-9

**Published:** 2020-07-03

**Authors:** Seri Jeong, Seom Gim Kong, Da Jung Kim, Sangjin Lee, Ho Sup Lee

**Affiliations:** 1 Department of Laboratory Medicine, Hallym University College of Medicine, 1, Singil-ro, Yeongdeungpo-gu, Seoul, 07441 South Korea; 2 Department of Pediatrics, Kosin University College of Medicine, 262, Gamcheon-ro, Seo-gu, Busan, 49267 South Korea; 3 Division of Hematology-Oncology, Department of Internal Medicine, Kosin University College of Medicine, 262, Gamcheon-ro, Seo-gu, Busan, 49267 South Korea; 4 Department of Statistics, Graduate School, Pusan National University, 2, Busandaehak-ro 63 beon-gil, Geumjeong-gu, Busan, 46241 South Korea

**Keywords:** Waldenström macroglobulinemia, Epidemiology, Incidence, Mortality, Survival

## Abstract

**Background:**

The epidemiological features of Waldenström macroglobulinemia (WM) have seldom been investigated at a national level, particularly in East Asia. The goal of our study is to present the incidence, prevalence, mortality, survival with competing risks, and causes of death of patients with WM.

**Methods:**

We used a national population-based database, operated by the Health Insurance Review and Assessment Service of the Korean government. This data includes information on all WM patients diagnosed according to uniform criteria, between 2003 and 2016.

**Results:**

The total number of patients newly diagnosed with WM during the study period was 427, with a male-to-female ratio of 3.2:1. The incidence increased from 0.03 to 0.10 per 10^5^ between 2003 and 2016, and the prevalence was 0.42 per 10^5^ in 2016. A total of 217 patients with WM died during the study period (standardized mortality ratio = 7.57), and the overall survival (OS) of WM patients was 47.5%. On multivariate analysis, older age was associated with worse OS (*P* <  0.0001). WM was the most common cause of death (*n* = 102, 48.6%), followed by other malignant neoplasms (*n* = 82, 39.0%).

**Conclusions:**

The national incidence of WM in Korea, a racially homogeneous country in Asia, was lower than that in previous reports from other countries, reflecting ethnic disparities. However, the incidence increased, and mortality was the highest ever reported. The main cause of death was WM in itself. This study reflects the need for greater awareness of WM, particularly in Asian countries.

## Background

Waldenström macroglobulinemia (WM) is an indolent B-cell malignancy characterized by the presence of immunoglobulin M (IgM) monoclonal gammopathy and lymphoplasmacytic bone marrow infiltration [1]. WM represents nearly 2% of all hematologic malignancies with 1000 to 1500 newly diagnosed cases per year in the United States based on the Surveillance, Epidemiology and End Results (SEER) database [2, 3].

Owing to the diverse clinical course of WM, treatment is not necessary for some patients at diagnosis. However, most patients with WM require systemic treatment at some time during the course of their disease [4]. The advent of new treatments, such as monoclonal antibodies, proteasome inhibitors, and Bruton tyrosine kinase inhibitors, with better understanding of the pathogenesis, have improved the expected clinical outcomes of WM [5, 6]. However, WM still remains incurable, and its management is becoming increasingly complex [3, 4].

According to population-based studies, the survival trends in patients with WM have improved within the last decade [7, 8]. However, variations in geographical location and ethnicity affect the survival of WM patients [8–10]. Other than in Europe and the United States, the epidemiological characteristics of WM have rarely been reported. In addition, only small numbers of Asian patients had been included in previously reported studies.

The present study was based on a comprehensive database, which is run by the National Health Insurance (NHI) of the Korean government. This database contains all the records of healthcare utilization among inpatients and outpatients. Therefore, this database was considered appropriate to reliably investigate the epidemiological features of WM.

Using this database, we performed a comprehensive population-based analysis to investigate the incidence, prevalence, mortality, survival with competing risks, and the causes of death in WM patients in the entire Korean population.

## Methods

### Data source

The data used in this study was extracted from the Health Insurance Review and Assessment Service (HIRA) database, which is based on data from a NHI system run by the Korean government. Healthcare institutions submit the medical data of all inpatient and outpatients in electronic format to the HIRA for reimbursement purposes. The claims data integrated by HIRA include all healthcare utilization information on inpatients and outpatients. Information regarding patient demographics, principal diagnosis, comorbidities, prescription history, and performed procedures is included in this database. The diagnostic information is based on the *International Classification of Diseases (ICD), 10th revision*. In this study, we obtained all WM cases from the HIRA database registered between January 2003 and the end of December 2016.

The causes of death for the deceased WM patients were also analyzed by linking the Statistics Korea data with the HIRA database. In the Statistics Korea data, the causes of death are documented according to the ICD-10, which are verified by physicians at the time of death and include information regarding all casualties. The number of person-years was calculated from the time of registration to death.

### Study population (patient selection)

All patients who had made claims to HIRA for WM from January 2003 to December 2016 were included in this study. The patients, who were diagnosed with WM based on criteria established by the NHI, were registered in the HIRA database. The criteria are similar to that of the criteria for WM described by Owen et al. [1]. The criteria for WM, defined by the ICD-10 code C88.0, are as follows: IgM monoclonal gammopathy, bone marrow infiltration by small lymphocytes, plasmacytoid cells and plasma cells, and typical immunophenotype (positive for surface immunoglobulin, CD19, CD20, and negative for CD5, CD10, and CD23). We excluded patients diagnosed before 2002 (*n* = 5) to minimize classification bias. The patients with non-IgM lymphoplasmacytic lymphoma and those with incomplete data (*n* = 33) were also excluded. Cases diagnosed only by death certificates were also excluded from the study. Since our study population depends on the ICD-10 code determined by physicians, it was difficult to completely rule out rare conditions such as IgM secreting marginal zone lymphoma and IgM plasma cell myeloma from WM.

The records of medical visits, demographic information, and death status were collected from the HIRA database for all patients diagnosed with WM. Patients included in this study were followed until the end of December 2016 to determine their vital status and causes of their deaths.

### Statistical analysis

We evaluated the incidence, prevalence, mortality, and survival rates of WM patients in Korea from 2003 to 2016. An incident case was defined as a patient who had been newly diagnosed and registered as having WM in the HIRA database during a corresponding year. A one-year washout period was applied to prevent prevalent cases from interfering with our data. Any patient free of any WM-related claims for at least one year in the HIRA database was defined as a new WM case in the washout period. The first date claimed in the HIRA database as a WM patient, was designated as the date of initial diagnosis. We calculated the incidence rate by dividing the number of total incident WM patients by the entire Korean population in the middle (mostly July) of each corresponding year.

A prevalent case was defined as a person who was registered in the HIRA database as a WM patient. Prevalent cases included all patients who had been diagnosed with WM during the corresponding year. Patients, who were registered as incident cases during previous years, were also included. The prevalence rates were calculated by dividing the number of total prevalent WM cases by the entire Korean population in each corresponding year. When calculating prevalence rates, those who died in the previous years were excluded from prevalent cases. The average age- and gender-specific incidence and prevalence rates were also calculated by dividing the number of WM cases in each specific age and gender group by the age- and gender-specific entire Korean population and averaging these data during the study period.

We calculated the annual mortality by dividing the number of total WM patients who died during that year by the entire number of WM patients in the HIRA database for the corresponding year. The mortality was compared with that of the general population listed in Statistics Korea using the SMR (standardized mortality ratio) with 95% CIs (confidence intervals). The SMR is defined as the ratio of patients who died from WM relative to the entire number of deaths in the general population. We calculated the SMR by dividing observed deaths by expected deaths derived from the mortality of the entire Korean population archived in Statistics Korea.

Survival information from the HIRA database was used in survival analysis. The Kaplan-Meier method with log-rank test was used to estimate the survival curves. The date of initial registration was regarded as the date of initial diagnosis. The person-years for WM patients were accumulated at the time of entry in the study until death. We also performed a subgroup analysis of survival by epoch of diagnosis, age, and treatment groups. Multivariate Cox proportional-hazards regression models were also used to examine the variables related to survival rates. The causes of death were analyzed for all WM mortality cases and presented according to the major disease classifications.

Statistical analyses were performed using R statistical software (version 3.4.4, R Foundation for Statistical Computing, Vienna, Austria) and SAS statistical analysis software (version 9.4, SAS Institute Inc., Cary, NC, USA). *P* values less than 0.05 were considered statistically significant.

## Results

### Incidence of WM

**Fig. 1:**
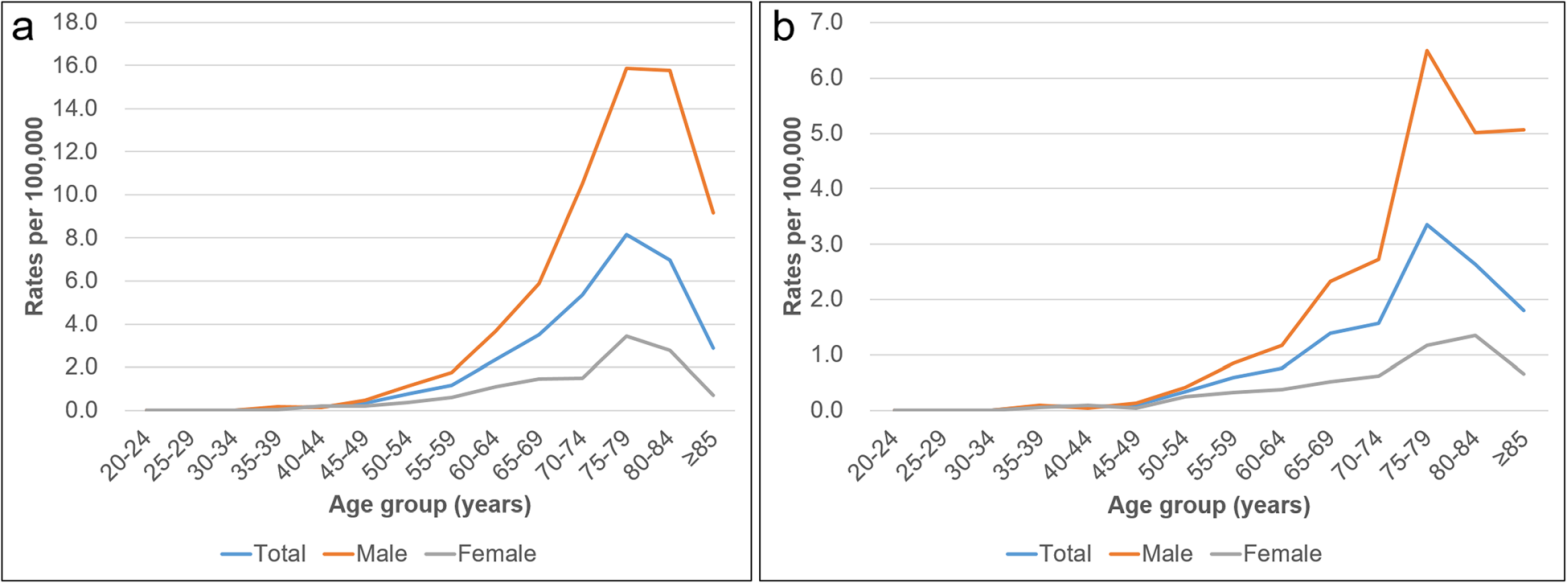
Incidence and prevalence of Waldenström macroglobulinemia by age and gender. Vertical axis shows incidence rates in 10^5^ people; horizontal axis presents age in 5-year increments until the age of 85 years. **a** Incidence **b** prevalence

The overall incidence and the age- and gender-specific annual incidence of WM are presented in Table 1 and Fig. 1a. The total number of patients with WM in Korea between 2003 and 2016 was 427, comprising 326 men and 101 women. The number of incident cases was 14 to 53, and the annual incidence increased from 0.03 to 0.10 per 100,000 people from 2003 through 2016. The median patient-age in these cases was 70.0 years (1st to 3rd quartile range: 61.0 to 76.0 years), and the median ages for men and women was 70.0 (1st to 3rd quartile range: 62.0 to 76.0 years), and 69.0 years (1st to 3rd quartile range: 58.0 to 76.0 years), respectively. The incidence rates by gender were 0.09 per 100,000 individuals for men and 0.03 per 100,000 individuals for women, rendering a male-to-female ratio of 3.2:1.

**Table 1:** Incidence and prevalence of Waldenström macroglobulinemia between 2003 and 2016

Year	Incident cases			Prevalent cases			Incidence per 10^5^/y^a^			Prevalence per 10^5^/y^b^		
	Total	Male	Female	Total	Male	Female	Total	Male	Female	Total	Male	Female
2003	14	8	6	19	12	7	0.03	0.03	0.02	0.04	0.05	0.03
2004	18	16	2	33	26	7	0.04	0.07	0.01	0.07	0.11	0.03
2005	15	13	2	45	36	9	0.03	0.05	0.01	0.09	0.15	0.04
2006	19	15	4	57	45	12	0.04	0.06	0.02	0.12	0.18	0.05
2007	21	20	1	66	55	11	0.04	0.08	0.00	0.13	0.22	0.04
2008	25	17	8	81	63	18	0.05	0.07	0.03	0.16	0.25	0.07
2009	28	22	6	94	71	23	0.06	0.09	0.02	0.19	0.28	0.09
2010	25	17	8	107	77	30	0.05	0.07	0.03	0.21	0.30	0.12
2011	39	28	11	132	99	33	0.08	0.11	0.04	0.26	0.39	0.13
2012	45	37	8	149	115	34	0.09	0.15	0.03	0.29	0.45	0.13
2013	37	27	10	162	122	40	0.07	0.11	0.04	0.32	0.48	0.16
2014	43	29	14	182	136	46	0.08	0.11	0.05	0.35	0.53	0.18
2015	45	36	9	202	151	51	0.09	0.14	0.03	0.39	0.59	0.20
2016	53	41	12	216	161	55	0.10	0.16	0.05	0.42	0.62	0.21
Total	427	326	101	1545	1169	376	0.06	0.09	0.03	0.22	0.33	0.11

^a^Incidence = (incident cases/total Korean population) × 10^5^

^b^Prevalence = (prevalent cases/total Korean population) × 10^5^

Age-specific incidence is shown in Fig. 1a. Among all age groups, the highest incidence rate of WM was observed in people aged between 75 and 79 years (8.15 per 100,000 individuals), accounting for 19.7% of all incident cases. The highest incidence peak for men (from 75 to 79 years old) was similar to that for women (Additional file 1: Table S1).

### Prevalence of WM

The annual prevalence and age- and gender-specific prevalence of WM are shown in Table 1 and Fig. 1b. The crude prevalence was 0.42 per 100,000 individuals, when calculated with the 2016 population as the denominator. The prevalence for men and women in 2016 were 0.62 and 0.21, respectively; the male-to-female ratio was 2.9:1, which was similar to that of incidence (Additional file 2: Table S2). On the other hand, the prevalence in 2003 was 0.04 per 100,000 individuals, with an 11.4-fold increase during the study period (13.4-fold for men and 7.9-fold for women). The annual increase rate between 2003 and 2016 was 18.96% (20.38% for men and 15.86% for women).

As shown in Fig. 1b, the shape of the prevalence plot was comparable to that of the incidence plot. The prevalence peak occurred at ages 75 to 79, accounting for 22.7% of all prevalent cases (Additional file 2: Table S2). The similar age group also exhibited the highest prevalence for both males and females.

### Mortality

In total, 217 of the 427 incident WM patients comprising 169 males and 48 females, died during the study period. The overall average annual mortality was 126.68 per 1000 individuals; 131.42 per 1000 males, and 116.38 per 1000 females. The age- and gender-specific SMRs for WM are shown in Table 2. The SMR for all WM patients was 7.57 (95% CI, 6.56 to 8.57), indicating that mortality in WM patients was significantly higher compared to that of the general population. The SMR for females (9.99; 95% CI, 7.16 to 12.82) was higher than that of males (5.56; 95% CI, 4.72 to 6.39).

**Table 2:** Age- and gender- specific standard mortality ratios and mortality of Waldenström macroglobulinemia

Age (years)	Total			Male			Female		
	O	E	SMR (95% CI)	O	E	SMR (95% CI)	O	E	SMR (95% CI)
20–49	1	0.18	5.46 (0.27–26.93)	1	0.15	6.47 (0.32–31.91)	0	0.04	0.00 (−)
50–59	8	1.14	7.03 (3.27–13.35)	5	1.21	4.12 (1.51–9.13)	3	0.16	18.32 (4.66–49.87)
60–69	39	4.34	8.99 (6.48–12.17)	29	5.10	5.69 (3.88–8.06)	10	0.50	20.02 (10.17–35.68)
70–79	75	13.92	5.39 (4.27–6.72)	60	15.85	3.78 (2.91–4.84)	15	1.88	7.97 (4.63–12.85)
> 80	94	9.11	10.32 (8.39–12.57)	74	8.09	9.14 (7.23–11.41)	20	2.22	9.03 (5.67–13.69)
Total	217	28.68	7.57 (6.56–8.57)	169	30.41	5.56 (4.72–6.39)	48	4.80	9.99 (7.16–12.82)

*O* number of observed deaths, *E* number of expected deaths, *SMR* standard mortality ratio, *CI* confidence interval

### Survival

**Fig. 2:**
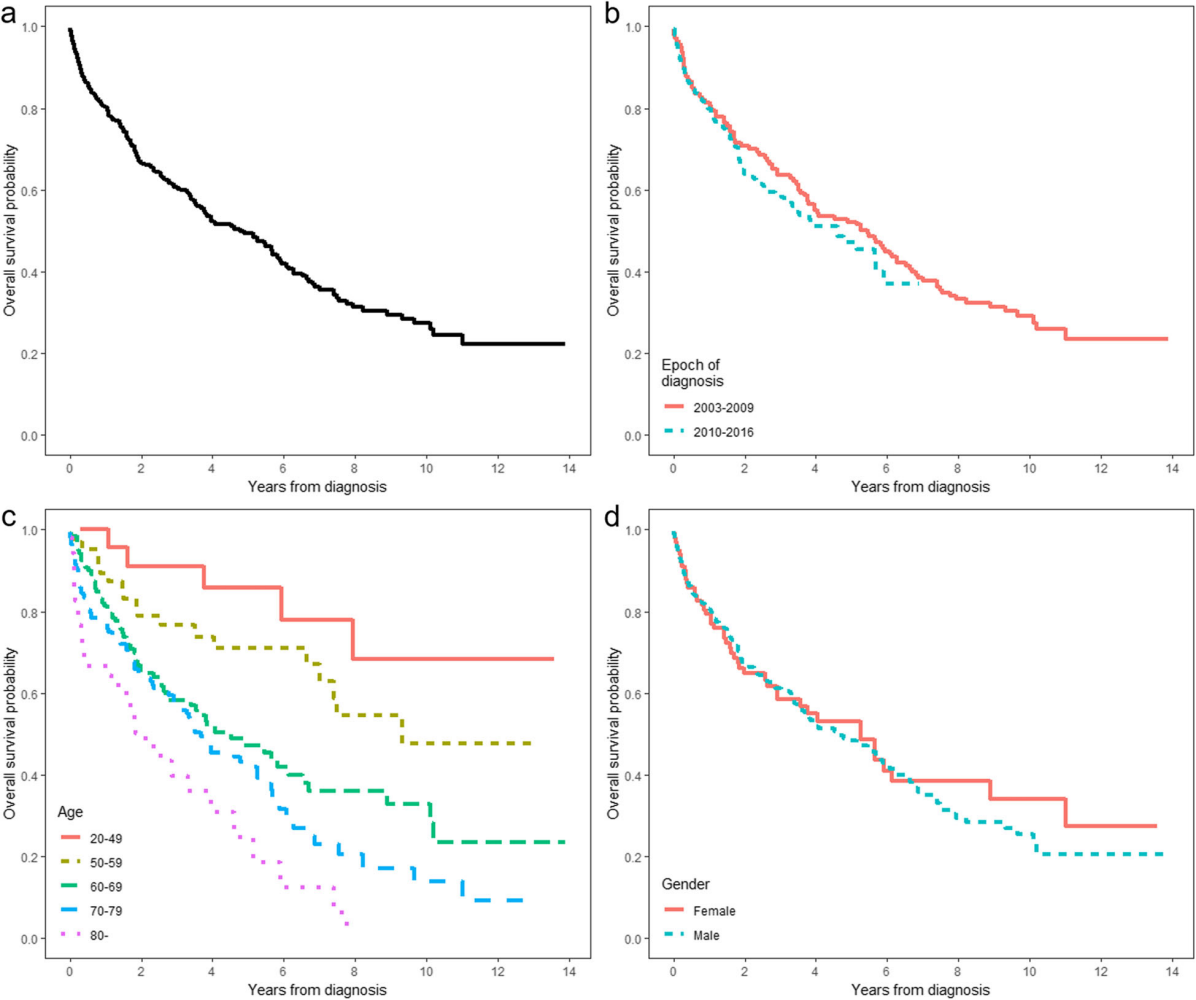
Overall survival in patients with Waldenström macroglobulinemia. **a** entire cohort **b** epoch of diagnosis **c** age categories **d** gender

The survival curve for WM patients is illustrated in Fig. 2. We followed 427 incident cases of WM from the time of diagnosis until December 31, 2016, which is equivalent to a total of 1368 person-years. In total, 217 incident cases died after a median follow-up of 4.5 years. The 14-year overall survival (OS) rate of WM patients was 47.5% (46.6% for males and 50.5% for females). The median OS of them was 4.5 years (95% CI, 3.6 to 5.5 years). The 2-, 5-, and 10-year survival rates were 69.0, 57.1, and 48.3%, respectively (Fig. 2a). For the 2003 to 2009 cohort, the median OS was 5.4 years (95% CI, 3.7 to 6.5 years). Meanwhile, the median OS was estimated 3.9 years (95% CI, 3.0 to 5.2 years) for the 2010 to 2016 cohort (Fig. 2b). There were statistical differences in the OS based on age (*P* <  0.01). The median OS for patients aged between 50 and 59, 60 and 69, 70 and 79, and ≥ 80 years were 9.3 years, 4.1 years, 3.5 years, and 1.8 years, respectively (Fig. 2c). The median OS according to the gender was 5.19 years for female, and 4.3 years for male (Fig. 2d). The specific chemotherapy agents administered to Korean patients are shown in Additional file 3: Table S3.

### Multivariate analysis for competing risks

On multivariate analysis for OS (Table 3), the factor significantly related to worse OS was older age (*P* <  0.01). The total hazard ratios (HR) for WM patients aged between 50 and 59, 60 and 69, 70 and 79, and ≥ 80 years, compared with patients between 20 and 49 years, were 2.30 (*P* = 0.10), 4.34 (*P <* 0.01), 5.82 (*P* < 0.01), and 9.57 (*P* < 0.01), respectively. In contrast, the gender (*P* = 0.94), and epoch of diagnosis (*P* = 0.35) were not significantly associated with OS.

**Table 3:** Multivariate analysis for overall survival in patients with Waldenström macroglobulinemia

Variable	Number (%)	2-year HR	95% CI	*P*	5-year HR	95% CI	*P*	10-year HR	95% CI	*P*	Total HR	95% CI	*P*
Age (years)													
20–49	26 (6.09)	Reference			Reference			Reference				Reference	
50–59	63 (14.75)	2.61	0.58–11.78	0.21	2.43	0.70–8.48	0.16	2.22	0.83–5.89	0.11	2.30	0.86–6.12	0.10
60–69	121 (28.34)	4.99	1.20–20.67	0.03	5.28	1.65–16.93	0.01	4.06	1.62–10.13	0.00	4.34	1.74–10.84	< 0.01
70–79	165 (38.64)	5.32	1.29–21.86	0.02	5.90	1.86–18.74	0.00	5.49	2.22–13.56	0.00	5.82	2.36–14.39	< 0.01
> 80	52 (12.18)	8.21	1.92–34.99	0.00	9.46	2.87–31.14	0.00	8.98	3.48–23.17	0.00	9.57	3.70–24.72	< 0.01
Gender													
Female	101 (23.65)	Reference		Reference				Reference			Reference		
Male	326 (76.35)	0.89	0.60–1.32	0.57	0.98	0.69–1.40	0.93	0.99	0.72–1.36	0.94	0.99	0.72–1.36	0.94
Epoch of diagnosis													
2003–2009	140 (32.79)	Reference		Reference				Reference			Reference		
2010–2016	287 (67.21)	1.15	0.79–1.68	0.47	1.10	0.80–1.50	0.56	1.15	0.85–1.55	0.36	1.15	0.85–1.56	0.35

*HR* hazard ratio, *CI* confidence interval

### Causes of death

The causes of deaths of WM patients are summarized in Table 4. WM-related deaths were recorded as the most common cause, accounting for 102 cases (48.57%). Malignant neoplasms constituted the second most common cause of death, being responsible for 82 deaths (39.05%). Among the 82 deaths due to malignant neoplasms, 23 were due to non-follicular lymphoma, and 22 were due to malignant plasma cell neoplasms.

**Table 4:** Cause of death among patients with Waldenström macroglobulinemia in Korea

Condition (ICD-10 code)	Male	Female	2003–2009	2010–2016	Total^a^ (%)
Neoplasm					
Malignant neoplasm (C00-C97 except for C88)	63	19	33	49	82 (39.05)
Waldenström macroglobulinemia (C88)	83	19	54	48	102 (48.57)
Other neoplasm (D00-D48)	1	0	0	1	1 (0.48)
Hematology (D50-D89)	2	1	1	2	3 (1.43)
Circulatory system					
Cardiovascular (I00-I52)	2	2	1	3	4 (1.90)
Cerebrovascular (I60-I69)	1	1	0	2	2 (0.95)
Infection (A00-B99)	0	2	1	1	2 (0.95)
Respiratory system (J00-J99)	4	1	2	3	5 (2.38)
Digestive system (K00-K93)	1	0	0	1	1 (0.48)
Musculoskeletal system and connective tissue (M00-M99)	1	0	1	0	1 (0.48)
Trauma (S00-S99, T00-T98, V01-V99, W00-W99, X00-X99, and Y00-Y98)	2	0	1	1	2 (0.95)
Other (R00-R99, and Z00-Z99)	5	0	2	3	5 (2.38)
Total	165	45	96	114	210 (100.00)

^a^A total of 7 out of 217 patients who died from Waldenström macroglobulinemia had unknown or unspecified causes

## Discussion

WM is an uncommon B-cell non-Hodgkin lymphoma, on which few epidemiological studies have been performed. In this study, the average 14-year incidence was 0.06 per 100,000 people. The incidence was stable between 1988 and 2005, at 0.3 per 100,000 people in the United States, and 0.55 per 100,000 people between 1999 and 2001 in England, which were significantly higher than the values found in this study [2, 11]. Differences in ethnicity, and geographic variations, exert a considerable influence on the lower incidence of WM in Korea. According to a previous report based on the SEER database, the incidence was higher in whites (0.41 per 100,000 people) than in nonwhites (0.20 per 100,000 people), suggesting distinct geographic differences [12]. Additionally, similar to the results of our study, the incidence of WM in Japan (0.043 per 100,000 person-years) and Taiwan (0.031 per 100,000 person-years) between 1996 and 2003, were lower than those of Asian ethnic groups (0.10 to 0.32) based on SEER data [13, 14]. These reports provide evidence that racial differences exist in the incidence of WM, and that environmental and/or genetic factors may have an influence on the development of WM. These results are also supported by the fact that the prevalence of IgM-monoclonal gammopathy of undetermined significance (MGUS), which is a precursor state to WM, is lower in the Asian population than in the other races [15, 16]. Furthermore, genetic predisposition, and immunologic factors in the pathogenesis of WM might be the reason behind these low incidence rates [17–21]. Although Korea may indeed have lower incidence, it should be noted that direct comparison with these studies may not be feasible. The incidence may be affected by the data collection methods and with the lapse of time. Additionally, the diagnostic criteria applied for WM may have been influenced by the cases diagnosed in the previous epoch [2, 4]. The period of our study covered the period during which the consensus panel recommendations for the diagnostic criteria of WM, published in 2003, were applied [1].

The annual incidence of WM has increased from 0.03 to 0.10 per 100,000 people in our study, compared with the consistent incidence reported by previous studies [2, 11, 22]. There are some studies, which showed that geographic alterations and racial differences are associated with significant increase in annual percentage change [12, 23]. This increase would partly be because of the increasing availability of diagnostic tools, and established criteria, which allowed the diagnosis of WM cases that would not have been possible to diagnose in the previous epoch. In addition, the differences between countries, in terms of the sample size, the growth rate of the aging population, and exposure to environmental factors, might explain these phenomena [12, 23]. Based on the incidence data, a comparatively lower prevalence rate than that of other countries was expected. However, the comparison was impossible because, to the best of our knowledge, no reports for the prevalence of WM were available.

Male dominance has been continuously reported in WM. In our study, the male-to-female ratio was 3.2:1, which is slightly higher than that reported in other studies from the Europe and United States (1.7 to 2.4:1) [11, 12, 14, 24, 25]. This study revealed the vulnerability of males in Korea to WM. The male-to-female ratios for WM in Japan (3.1:1) [23] and in Olmsted County (3.1:1) [22] were similar to our value, suggesting regional differences. In regard to the age distribution, the incidence peak occurred in people aged between 75 and 79 years, which is consistent with the values reported for other countries [12, 25].

The SMR of WM patients in our study (7.57) was the highest ever reported. A recent study, conducted in Olmsted County, presented a SMR of 2.4 for the patients diagnosed with WM after 2000 [22]. Another study reported that the SMR of asymptomatic WM was equivalent to that of the general population, while that of symptomatic patients was 5.4 [26].

In respect of survival, the median OS of Korean WM patients (4.5 years; 95% CI, 3.6 to 5.5 years) was significantly lower than that of Asians living in United States (7.4 years for patients diagnosed with WM between 2003 and 2009; 95% CI, 6.8 to 8.2 years), implicating an environmental effect [9]. Considerably longer median OS (7 to 25 years) has been reported from the Europe and United States owing to ethnic disparities, epoch of diagnosis, and different study designs, such as hospital-based cohort and population-based research [4, 9, 10, 27, 28]. The OS of our study (47.5%) was also lower than the range reported in previous studies (between 57 and 62% in England and the United States) [2, 4, 11]. The OS in our study was substantially worse than those of other countries, taking into account the epoch of diagnosis, because the study periods of the others were mostly before 2010.

Although there is no standard of care established for WM, the advent of anti-CD20 monoclonal antibodies, nucleoside analogs, alkylating agents, and proteasome inhibitors have shown high response rates and have resulted in better OS [3, 29, 30]. In particular, rituximab singly or in combination has been commonly used as first-line therapy, and has contributed to better OS (62 to 97.1%) in the Western countries [31–34]. However, the HIRA does not reimburse the rituximab-based chemotherapy because of the paucity of studied data for WM patients in Korea. Exclusion from the HIRA system could therefore be the cause for the higher SMR and the poorer survival of Korean patients with WM. It would be necessary to add at least rituximab-based treatment to the reimbursement system of HIRA for improving the outcome of WM patients.

Additionally, we investigated the trends of the mortality rates in the general population to find out the reason why the OS did not improve according to the epoch of diagnosis. However, there were no notable differences of general population which could influence on the mortality of WM patients. The restrictions in the appropriate prescription of rituximab combined chemotherapy owing to reimbursement issues could have contributed to the lack of improvement. Furthermore, a relatively short follow-up period, considering the expected longer life span of WM patients [27], in addition to an inevitable small sample size compared to other population-based studies [4, 7], may have affected these survival results.

Our competing risk analysis showed that age was a strong factor (*P* < 0.01) for OS in patients with WM. The significant association between old age and poorer outcome was persistently reported in previous studies [2, 4, 11].

The main cause of death in our cohort was directly attributable to WM, and the magnitude (48.57%) was almost two times higher than that of a previous report from the SEER database (25%) [4]. The poorer survival of Korean patients owing to obstacles in the reimbursement system to newly introduced therapies could be associated with higher WM-related deaths. The next most common cause of death was non-follicular lymphoma, which was consistent with the higher proportion of deaths due to lymphoma in the SEER database [4]. The third most common cause was malignant plasma cell neoplasms, implicating considerable relevance to WM [35]. Because our data based on the records archived in Statistics Korea, it was difficult to differentiate non-follicular lymphoma, and plasma cell neoplasm from WM. The possibility of misclassification should be considered.

This study had several limitations. The lack of detailed clinical information such as the symptoms, the laboratory data, or genetic features, led to restrictions on the adjustments of severity for each WM patient. Moreover, classification bias could exist because we used registry data based on physicians’ diagnoses without additional pathological confirmation. Despite these limitations, the strength of our study is the use of a nationwide population database of recent WM patients, after application of established diagnostic criteria. To the best of our knowledge, no other study has reported on the incidence, prevalence, mortality, and causes of death using a recent and nationwide data source, especially in Asia. The relatively large sample size covering the entire national population and unbiased measures used in this study could provide reliable information on WM patients.

## Conclusions

In conclusion, we are the first to report the epidemiologic features of WM based on the recent nationwide database of Korea, a racially homogeneous country of East Asia. This study confirmed the low incidence of WM in Korea, reflecting ethnic disparities. However, the incidence increased three fold between 2003 and 2016, and mortality was the highest ever reported. The most common cause of death was directly attributable to WM. This study indicates that more attention needs to be paid to WM patients, particularly in Eastern Asian countries.

## Supplementary information

**Additional file 1: Table S1.** Age-specific annual incidence of Waldenström macroglobulinemia between 2003 and 2016.

**Additional file 2: Table S2.** Age-specific prevalence of Waldenström macroglobulinemia in Korea, 2016.

**Additional file 3: Table S3.** The specific chemotherapy agents administered to Korean patients with Waldenström macroglobulinemia.

## Data Availability

The datasets limited to anonymisable information and supporting the conclusions of this article are included within this article (Tables, Figures, and Additional files). The repository data for public release is not available because of the personally identifiable information. The full dataset includes clinic centers in which they attend, insurance conditions. Therefore, concerning privacy risks, the data is managed by authorized executive supervisor. If one researcher asks to access data, the researcher should submit the security memorandum and pledge to the Institutional Review Board of National Health Insurance Sharing Service. After approval, the person in charge releases data with blind identification for the discrete requirements and the data should be analyzed only in permitted rooms in centers of National Health Insurance Service. The other researchers could access these data in the same manner as the authors and the authors did not have any special access privileges. Contact information for a data access committee is listed as follows: National Health Insurance Sharing Service, Tel: 82–33–736-2432; Official internet site: https://nhiss.nhis.or.kr/bd/ay/bdaya001iv.do.

## Abbreviations

CI: Confidence interval
HIRA: Health Insurance Review and Assessment Service
HR: Hazard ratio
ICD: International Classification of Diseases
IgM: Immunoglobulin M
MGUS: IgM-monoclonal gammopathy of undetermined significance
OS: Overall survival
SEER: Surveillance, Epidemiology and End Results
SMR: Standardized mortality ratio
WM: Waldenström macroglobulinemia
